# Haemoprotozoa: Making biological sense of molecular phylogenies

**DOI:** 10.1016/j.ijppaw.2017.08.007

**Published:** 2017-08-26

**Authors:** Peter O'Donoghue

**Affiliations:** Department of Parasitology, University of Queensland, Brisbane 4072, Queensland, Australia

**Keywords:** Haemoprotozoa, Phylogeny, Biology, Vertebrate hosts, Invertebrate vectors

## Abstract

A range of protistan parasites occur in the blood of vertebrates and are transmitted by haematophagous invertebrate vectors. Some 48 genera are recognized in bood primarily on the basis of parasite morphology and host specificity; including extracellular kinetoplastids (trypanosomatids) and intracellular apicomplexa (haemogregarines, haemococcidia, haemosporidia and piroplasms). Gene sequences are available for a growing number of species and molecular phylogenies often link parasite and host or vector evolution. This review endeavours to reconcile molecular clades with biological characters. Four major trypanosomatid clades have been associated with site of development in the vector: salivarian or stercorarian for *Trypanosoma*, and supra- or peri-pylorian for *Leishmania*. Four haemogregarine clades have been associated with acarine vectors (*Hepatozoon* A and B, *Karyolysus, Hemolivia*) and another two with leeches (*Dactylosoma, Haemogregarina* sensu stricto). Two haemococcidian clades (*Lankesterella, Schellackia*) using leeches and mosquitoes (as paratenic hosts!) were paraphyletic with monoxenous enteric coccidia. Two major haemosporidian clades have been associated with mosquito vectors (*Plasmodium* from mammals, *Plasmodium* from birds and lizards), two with midges (*Hepatocystis* from bats, *Parahaemoproteus* from birds) and two with louse-flies and black-flies (*Haemoproteus* and *Leucocytozoon* from birds). Three major piroplasm clades were recognized: one associated with transovarian transmission in ticks (*Babesia* sensu stricto); one with pre-erythrocytic schizogony in vertebrates (*Theileria/Cytauxzoon*); and one with neither (*Babesia* sensu lato). Broad comparative studies with allied groups suggest that trypanosomatids and haemogregarines evolved first in aquatic and then terrestrial environments, as evidenced by extant lineages in invertebrates and their radiation in vertebrates. In contrast, haemosporidia and haemococcidia are thought to have evolved first in vertebrates from proto-coccidia and then incorporated invertebrate vectors. Piroplasms are thought to have evolved in ticks and diversified into mammals. More molecular studies are required on more parasite taxa to refine current thought, but ultimately transmission studies are mandated to determine the vectors for many haemoprotozoa.

## Highlights

• Molecular phylogenies mostly reconcile with conventional classifications for haemoprotozoa.
• Clades correlate well with parasite developmental stages, vertebrate hosts and invertebrate vectors.
• Trypanosomes, haemogregarines and piroplasms appear to have evolved in aquatic invertebrates.
• Haemosporidia and haemococcidia appear to have evolved in vertebrates from proto-coccidia.

## Graphical abstract

**Figure:**
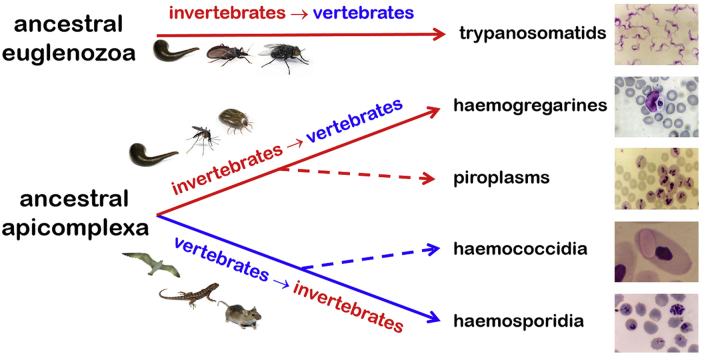

## 1 Introduction

The determination of phylogenetic relationships between organisms has changed radically over the last few decades. Modern molecular phylogenies have taken precedence over conventional biological phylogenies because genotypic differences are thought to better reflect evolutionary relationships than phenotypic comparisons (cf. Zimmer and Emlen, 2013.). However, contemporary studies should endeavour to include prior knowledge to provide a better contextual framework for understanding. In science, new information supports, refutes, extends, supplants or is compared to established knowledge. Many molecular phylogenies have been published for a range of protozoan parasites, but they are not always placed in biological context. Haemoprotozoan parasites are unique unicellular protists that have developed sophisticated life-cycles alternating between development in the tissues and blood of a range of vertebrate hosts and the gut and tissues of various blood-feeding invertebrate vectors. The purpose of this article is to reconcile relationships gleaned from modern gene analyses with the rich history of information derived from conventional studies; involving not only the haemoprotozoan parasites themselves but also their vertebrate hosts and invertebrate vectors. It is an attempt to make biological sense of molecular phylogenies.

Taxonomic classification systems have used a constellation of characters to group organisms according to their differences/similarities, theoretically reflecting their kinship and ancestry. For centuries, multiple phenotypic characters have been used to identify several groups of haemoprotozoan parasites differing in host occurrence, range, parasite morphology, development, transmission cycles, biochemical and physiological characteristics (especially pertinent to parasite nutrition and culture) and immunological interactions with their hosts (Levine et al., 1980, Lee et al., 1985, Lee et al., 2000). More recently, the molecular biology revolution has allowed many additional genotypic characters (notably protein and DNA sequences) to be used for molecular taxonomy, population genetics, phylogenetic analyses and even molecular mining (cf. Loker and Hofkin, 2015). Indeed, molecular studies have completely revolutionized the way we look at eukaryotes. Rather than representing one cohesive group defined by the possession of a membrane-bound nucleus, molecular phylogenetic studies have revealed the existence of numerous phyla belonging to at least five super-groups (Adl et al., 2012):

• opisthokonts (stages with single posterior flagellum, including animals, fungi and choanoflagellates);
• amoebozoa (with non-eruptive pseudopodia, including lobose amoebae and slime molds);
• excavata (with ventral feeding groove, including metamonads, euglenozoa and heterolobosea);
• archaeplastids (with chloroplasts, including land plants, green and red algae); and
• SAR [stramenopiles-alveolata-rhizaria] (with heterokont flagella such as the opalinids; or with cortical alveoli such as the apicomplexans, ciliates and dinoflagellates; or with filopodia or reticulopodia such as the ascetospora, foraminifera and radiolaria).

In the context of this paper, members of three different supergroups are involved: kinetoplastid haemoflagellates belonging to the Euglenozoa in the supergroup Excavata; haemosporozoan parasites belonging to the Apicomplexa in the SAR supergroup; while all their vertebrate hosts and invertebrate vectors belong to the supergroup Opisthokonta.

## 2 Haemoprotozoa

**Fig. 1:**
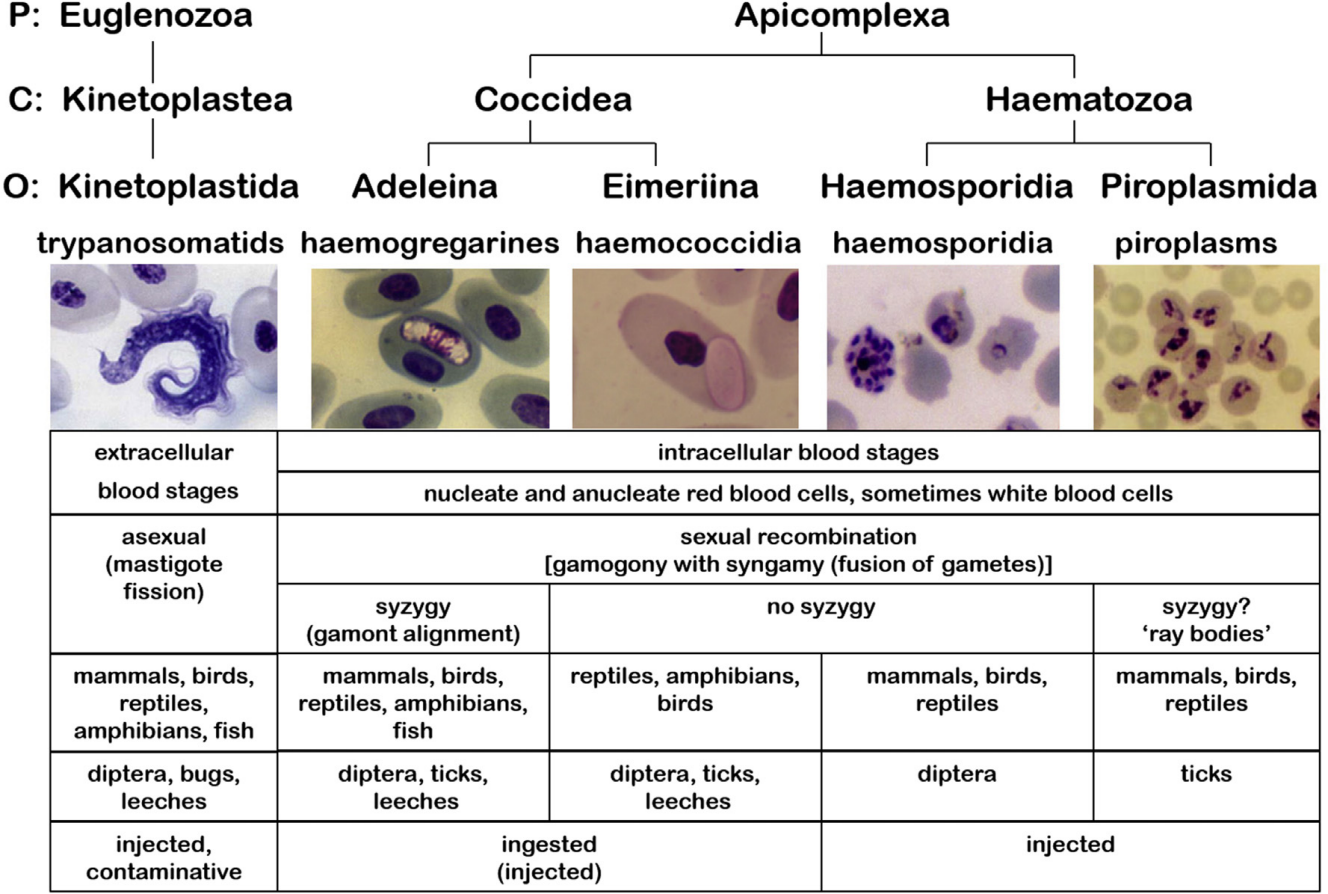
Key characteristics of the five haemoprotozoan assemblages.

Conventionally, five groups of haemoprotozoan parasites are recognized (cf. Lee et al., 2000) (Fig. 1): one group of euglenozoan flagellates, trypanosomatid kinetoplastids, which form extracellular developmental stages (trypomastigotes) in host blood plasma and undergo asexual reproduction by binary fission (some representatives also form intracellular amastigote stages in host tissues); and four groups of apicomplexan parasites, which form intracellular developmental stages within vertebrate blood cells and undergo both asexual and sexual reproduction, the latter involving the formation of gametes which subsequently fuse (syngamy) with or without paired alignment (syzygy). The four apicomplexan groups include the haemogregarines (adeleorine coccidia) exhibiting syngamy with syzygy, the haemococcidia (eimeriine coccidia) exhibiting syngamy without syzygy, the haemosporidia (pleomorphic haematozoa) exhibiting syngamy without syzygy, and piroplasms (pyriform haematozoa) exhibiting syngamy possibly with syzygy (via pairing of unique ray-bodies).

By definition, the host in which sexual reproduction of the parasite occurs is called a definitive host, the host in which only asexual development occurs is called an intermediate host, and a host in which no further parasite development occurs is a paratenic (transport) host. Because haemoprotozoan parasites cycle between vertebrate and invertebrate hosts, it is important to determine which hosts act as definitive or intermediate hosts. The five haemoprotozoan groups exhibit considerable variability in their vertebrate host range and specificity; many parasite species being oioxenous (highly host-specific) for particular host species, while others are stenoxenous for multiple vertebrate hosts (usually closely-related species). Infections have been recorded in most classes of vertebrates; in fish, amphibians, reptiles, birds and mammals (including primates). Vector range and specificity is also quite variable, ranging from insects to arachnids to leeches. Trypanosomatids have been shown to be transmitted by insects (flies, bugs) and leeches; haemogregarines and haemococcidia by insects, ticks and leeches; haemosporidia by insects (especially mosquitoes); and piroplasms by ticks. All these vectors are haematophagous, i.e. they feed on blood from vertebrate hosts. Transmission of parasites from vertebrate hosts to vectors occurs when the invertebrates use specialized mouthparts to penetrate epidermal layers and feed on blood, either by sucking or pool-feeding. Parasites taken up during feeding often then undergo further development in organs of the vectors. Transmission of parasites from vectors to vertebrate hosts occurs via three basic mechanisms: inoculative (parasites injected by vector along with salivary secretions containing anti-coagulants and other vaso-active substances to assist feeding; route involved in salivarian transmission); contaminative (parasites voided by vector contaminate wound site; route involved in stercorarian transmission); or consumptive (whereby the infected vector is eaten by the vertebrate host; route somewhat analogous to predator-prey transmission). Using multiple and varied differences in host, vector and parasite biology as taxonomic characters, a staggering number of genera and species of haemoprotozoan parasites have been identified: over 1740 species belonging to some 48 genera.

## 3 Evolutionary origins

### 3.1 Speculation

Scientists working on this tripartite interaction of protist parasite, invertebrate vector and vertebrate host have often contemplated the evolutionary origins of the haemoprotozoan groups, especially since many of them seemingly have analogues that may be free-living, commensal or parasitic (although not in blood). In particular, scientists often ask the question: which host came first? The vertebrate host or the invertebrate vector? Logical scenarios have been postulated whereby free-living protist ancestors have been taken up by ancestral hosts to become monoxenous/homoxenous (one-host) parasites that have subsequently adopted blood-borne transmission to become dixenous/heteroxenous (two-host) parasites (Lee et al., 1985, Lee et al., 2000). Comparisons of conventional taxonomic characters (such as protist morphology, development, biology, host range, specificity) have led to two schools of thought. Some haemoparasites (haemococcidia, haemosporidia) are thought to have first evolved in vertebrate hosts from gut parasites (cf. Garnham, 1966), while others (trypanosomes, haemogregarines, piroplasms) are thought to have first parasitized invertebrate hosts, some becoming blood-feeders (cf. Hoare, 1972). Traditionally, scientists turn to the fossil record to seek corroboration for their evolutionary theories. Because blood protozoa are unicellular, microscopic organisms with ‘soft’ bodies (without tests, shells or skeletons), they were not thought to be represented in the fossil record, until recently.

### 3.2 Fossil records

**Fig. 2:**
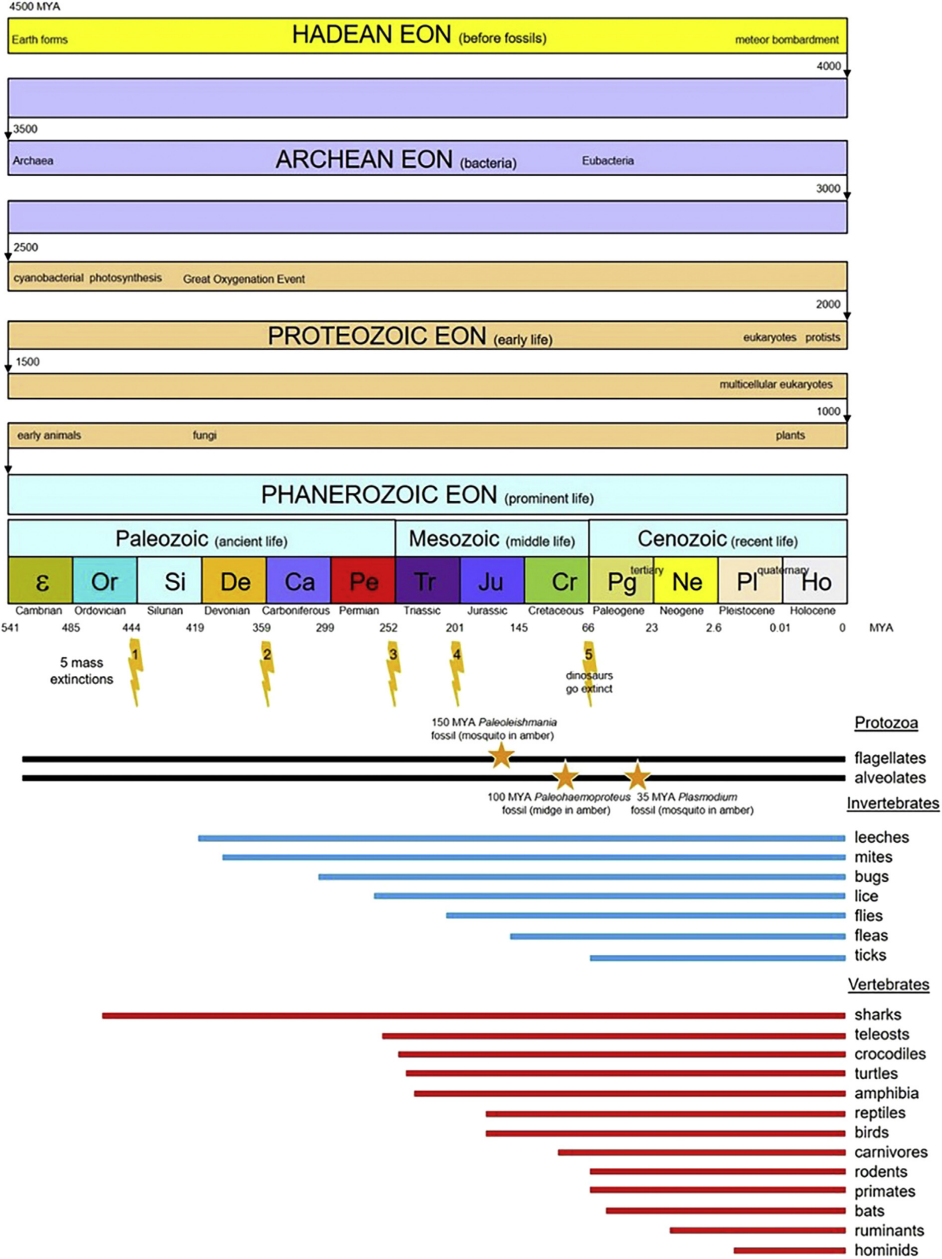
Geological time periods with milestones in the development of life on Earth, together with historical extent of fossil records for particular assemblages.

The ancestors of many vertebrates and some invertebrates are well-represented in the fossil record (Fig. 2). In the 4.5 billion year (BY) history of the planet Earth, the first 4 BY saw life emerge in the form of Archaea and Eubacteria in the Archean Eon (4.0–2.5 BY), then progress to early eukaryotes (protists, plants, fungi and animals) in the Proteozoic Eon (2.5–0.5 BY). The last 0.5 billion years has seen an explosion of life in the Phanerozoic Eon, from ancient to middle to recent life-forms in the Paleozoic, Mesozoic and Cenozoic Periods. The fossil record has shown 5 mass extinction events culminating in the extinction of the dinosaurs in the late Cretaceous Period some 65 million years ago (MYA). Fossils of vertebrate animals have been found throughout the Phanerozoic Eon from 460 to 1 MYA, including fossilized sharks from the Ordovician, teleosts and crocodiles from the Permian, turtles and amphibians from the Triassic, reptiles and birds from the Jurassic, carnivores from the Cretaceous, rodents, primates and bats from the Paleogene (Tertiary), ruminants from the Neogene (Tertiary), and hominids from the Pleistocene (Quaternary) Periods (cf. Benton and Harper, 2009).

Fossils of many invertebrate groups have also been found throughout the Phanerozoic Eon from 420 to 66 MYA; including fossilized leeches and mites from the Silurian, bugs from the Carboniferous, lice from the Permian, flies from the Triassic, fleas from the Jurassic and ticks from the Cretaceous Periods (cf. Taylor and Lewis, 2007). Protists have been found to be represented in the fossil record as early as the Cambrian Period some 540 MYA. Fossils have been found of free-living flagellates and ciliates which formed hardened tests, shells or endoskeletons (cf. Benton, 1993). More recently, microfossils have been found of haemoprotozoa in invertebrates which had become encased in amber: *Paleoleishmania* in a mosquito from the Jurassic 150 MYA (Poinar and Poinar, 2004), *Paleohaemoproteus* in a midge from the Cretaceous 100 MYA (Poinar and Telford, 2005), and *Plasmodium* in a mosquito from the Paleogene 35 MYA (Poinar, 2005). The estimated ages of the fossils of these ancestral haemoprotozoa and the ancestors of their current vertebrate and invertebrate host groups overlap considerably and there is no logical sequence to suggest which host group came first.

### 3.3 Molecular phylogeny

Over the last three decades, numerous molecular biological studies have succeeded in sequencing DNA extracted from different organisms and then comparing the sequences using various algorithms to infer phylogenetic relationships between organisms. A range of partial gene sequences have been examined for haemoprotozoan parasites; including nuclear (*ssurRNA*, *gGAPDH*, *hsp70*, *polA*, *rpoIILS*, *gp63*, *cpb*, *asl*, *ef2*, *cct7*, etc.), mitochondrial (*cytb*, *cox*) and apicoplast (*clpC*) genes. Numerous phylogenetic trees have been published showing evolutionary relationships between various haemoprotozoan assemblages, but often with little biological data given for those assemblages. Molecular analyses can be used not only to determine the extent of intra- and inter-specific variation (i.e. to characterize species, subspecies, strains, genotypes), but also to examine variation between higher taxonomic rankings (including genera and families). The latter approach was taken in this paper to look for phenotypic patterns in molecular phylogenies; that is, to compare old conventional and newly emergent data-sets, particularly looking for correlates or contradictions. This took the form of identifying molecular clades that also appeared to be monophyletic for particular phenotypic traits (especially focussing on parasite morphology, developmental cycle, host/vector range and specificity, where known). I have deliberately used the term clade rather than lineage to mean evolution by descent (dictionaries define a clade as a group of organisms believed to comprise all evolutionary descendants of a common ancestor, while lineage is defined as a sequence of species each of which is considered to have evolved from its predecessor). Obviously, such speculations about evolutionary patterns and clades are tentative and are by no means comprehensive (viz: inclusive of all species in that taxon) because molecular (and biological) data-sets are frequently restricted to particular ‘representative’ species. Nevertheless, comparative studies do facilitate critical review of prevailing concepts and provide direction and targets for future research.

## 4 Trypanosomes

### 4.1 Biodiversity

Several genera of trypanosomatid flagellates are found as blood parasites in vertebrates which are transmitted by invertebrate haematophagous vectors. They belong to an ancient lineage of mitochondriate eukaryotes where mitochondrial DNA (kDNA) is enclosed in an organelle called the kinetoplast which is separate from the nuclear DNA. Kinetoplastid flagellates have been classified within the phylum Euglenozoa in the supergroup Excavata (Adl et al., 2012). They are characterized by the possession of a kinetoplast, a flagellar pocket, basal bodies with three microtubular roots and paraxonemal (paraxial or paraflagellar) rods, and asexual multiplication by longitudinal binary fission (Hoare, 1972). Two major kinetoplastid groups are recognized: bodonids with two flagella (most being free-living bactivores in aquatic/terrestrial habitats); and trypanosomatids with a single flagellum (most being parasites of animals or plants with monoxenous or dixenous life-cycles).

The trypanosomes have been grouped together in the family Trypanosomatidae and comparative analyses of conventional phenotypic characters have suggested their evolution from free-living aquatic forms into aquatic invertebrates (annelids and leeches) with subsequent transmission to aquatic vertebrates (fishes and amphibians). It is thought some groups became parasitic in terrestrial insects (diptera and hemiptera) some 150 MYA where they exhibited monoxenous cycles maintained by contamination, necrophagy, coprophagy and predation (Hoare, 1972). Some groups then became parasitic in blood-feeding insects and were transmitted to terrestrial vertebrates (reptiles, birds and mammals) where they are maintained by stercorarian (contaminative) transmission between bugs and nesting vertebrates or by salivarian (inoculative) transmission between dipteran vectors and gregarious vertebrates. Kinetoplastids comprise robust and diverse groups ideal for testing phylogenetic hypotheses because they contain many extant species ranging from free-living forms to monoxenous parasites to dixenous parasites.

**Fig. 3:**
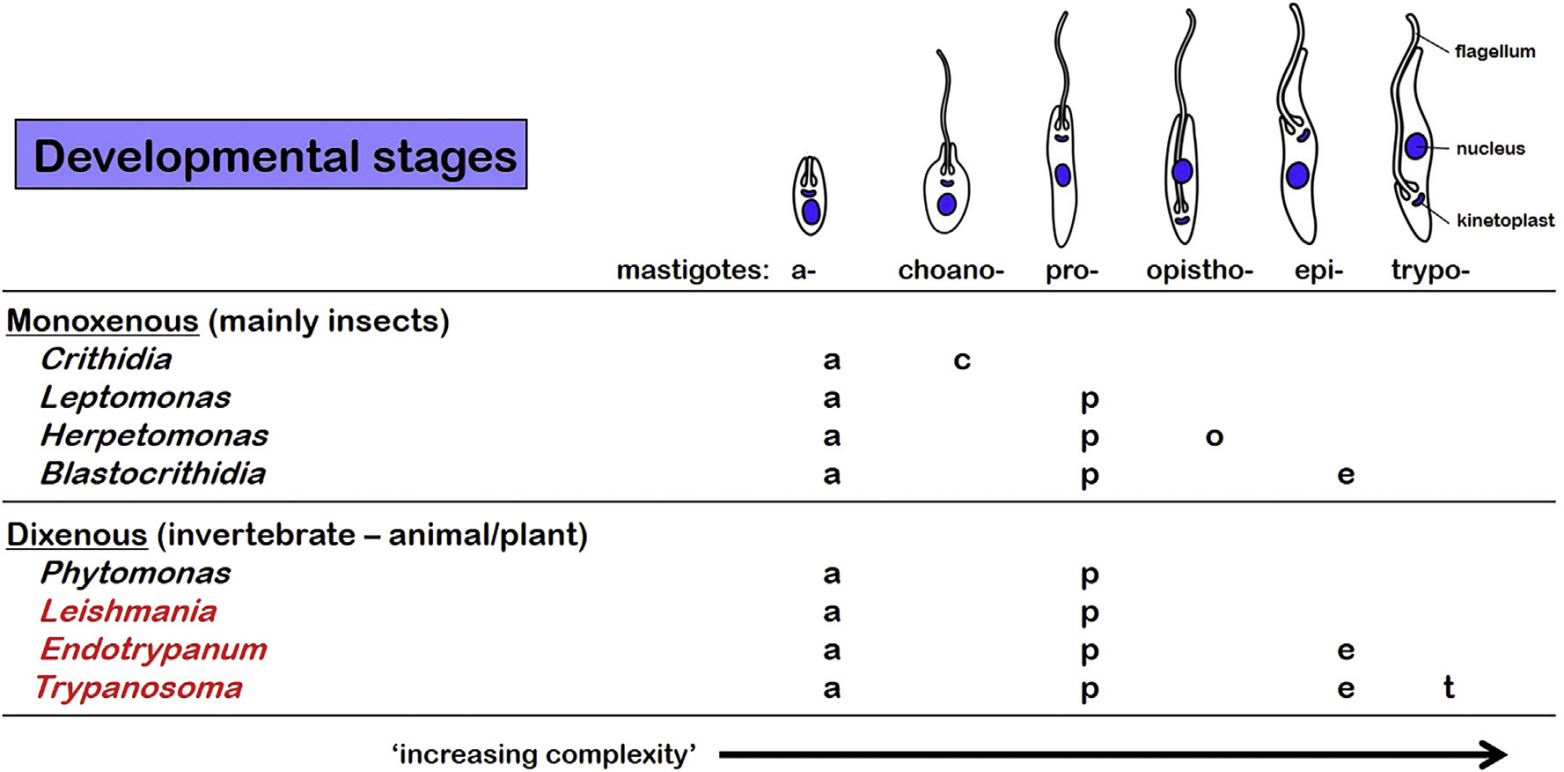
Developmental stages formed by kinetoplastid flagellates (blood-borne genera shown in red). (For interpretation of the references to colour in this figure legend, the reader is referred to the web version of this article.)

Kinetoplastids also exhibit increasing morphological/ultrastructural complexity in cellular organization (Fig. 3) which are thought to reflect evolutionary grades or clines. Amastigotes are simple non-flagellated cells, choano-, pro- and opistho-mastigotes are flagellated cells with elongate flagella, while epi- and trypo-mastigotes are flagellated cells with undulating membranes (Hoare, 1972). Most kinetoplastids have amastigote and promastigote developmental stages but monoxenous parasites of insects (such as *Crithidia*, *Leptomonas*, *Herpetomonas* and *Blastocrithidia*) do not have more elaborate forms whereas dixenous parasites of plants or animals with invertebrate vectors (such as *Phytomonas*, *Leishmania*, *Endotrypanum* and *Trypanosoma*) do have more morphologically complex forms such as epimastigotes and trypomastigotes (Lee et al., 1985, Lee et al., 2000).

Over 600 species of blood-borne kinetoplastids have been classified into four genera in two families on the basis of multiple phenotypic characters (host occurrence, vectors, transmission cycles, developmental stages, pathogenicity, culture requirements, etc.). They are all dixenous parasites which form asexual developmental stages (extracellular trypomastigotes and/or intracellular amastigotes) in vertebrate blood and luminal stages in their invertebrate vectors (flies, bugs and leeches). A total of 52 *Cryptobia* (*Trypanoplasma*) species have been identified in the family Bodonidae, and 10 *Sauroleishmania* species, 35 *Leishmania* species and 520 *Trypanosoma* species in the family Trypanosomatidae (Table 1). Species in the latter two genera are responsible for severe clinical infections in a range of vertebrate hosts, including humans, and are transmitted by arthropod and leech vectors.

**Table 1:** Genera of kinetoplastid haemoflagellates.

Assemblage	Genera	No. spp.	Vertebrate hosts	Invertebrate vectors
F: Trypanosomatidae	*Trypanosoma*	520	mammals, reptiles, frogs, birds, fish	arthropods, leeches
F: Trypanosomatidae	*Leishmania*	35	mammals	arthropods
F: Trypanosomatidae	*Sauroleishmania*	10	lizards/snakes	sandflies?
F: Bodonidae	*Cryptobia (Trypanoplasma)*	52	fish	leeches

### 4.2 Molecular clades

Recent molecular phylogenetic studies based on *ssurRNA* gene sequences by Lukes et al. (2014) have shown some monoxenic species in fish (*Ichthyobodo*) and amoebae (*Perkinsiella*) to have separated early from a broad clade containing most other kinetoplastids. The latter clade contains several groups of free-living genera (*Parabodo, Neobodo*, *Procryptobia*, *Bodo*, *Klosteria*, *Rhynchobodo*, *Rhynchomonas*, *Dimastigella*, *Phanerobia*, *Cruzella*, *Cryptaulax* and *Actuariola*) interspersed with monoxenous genera (*Cryptobia*, *Jarrellia*, *Azuminobodo* and *Paratrypanosoma* species from fish, whales, ascidians and mosquitoes respectively) and dixenous genera (*Trypanoplasma* from fish and most trypanosomatid genera). Their polyphyletic relationships suggest that parasitism in kinetoplastids did not have a single origin from free-living ancestors but appeared on several occasions within different host assemblages.

Studies focussing on parasitic trypanosomatid genera (Lukes et al., 2014) revealed most monoxenous genera (*Strigomonas* and *Angomonas* from mosquitoes, *Blastocrithidia* from bugs, *Sergeia* from midges, *Wallaceina* and *Herpetomonas* from flies, *Leptomonas* from insects, *Crithidia* from bees, *Blechomonas* from fleas) diverged more recently than dixenous genera (*Trypanosoma* with fly, bug or leech vectors, *Leishmania* with fly vectors, *Phytomonas* with bug vectors). This contradicts conventional dogma which assumes evolution towards increasing complexity because it suggests that parasitic trypanosomatids did not evolve from simple monoxenous to more complex dixenous life-cycles. Collective genomic studies conducted on parasitic trypanosomatid species have yielded consensus trees (Jackson, 2015) separating *Trypanosoma* species into two major clades; one comprising salivarian trypanosomes (e.g. *T. brucei, T. congolense, T. vivax*) and the other stercorarian trypanosomes (e.g. *T. cruzi, T. rangeli, T. grayi, T. theileri*). *Leishmania* species were grouped together in a separate clade with six main lineages*: L. australiensis; L. panamensis* and *L. braziliensis; L. tarentolae; L. mexicana; L. donovani* and *L. infantum;* and *L. major* (Asato et al., 2009) The *Trypanosoma* and *Leishmania* clades were well separated by other monoxenous (*Angomonas, Strigomonas, Crithidia, Leptomonas*) and dixenous (*Phytomonas*) species.

**Fig. 4:**
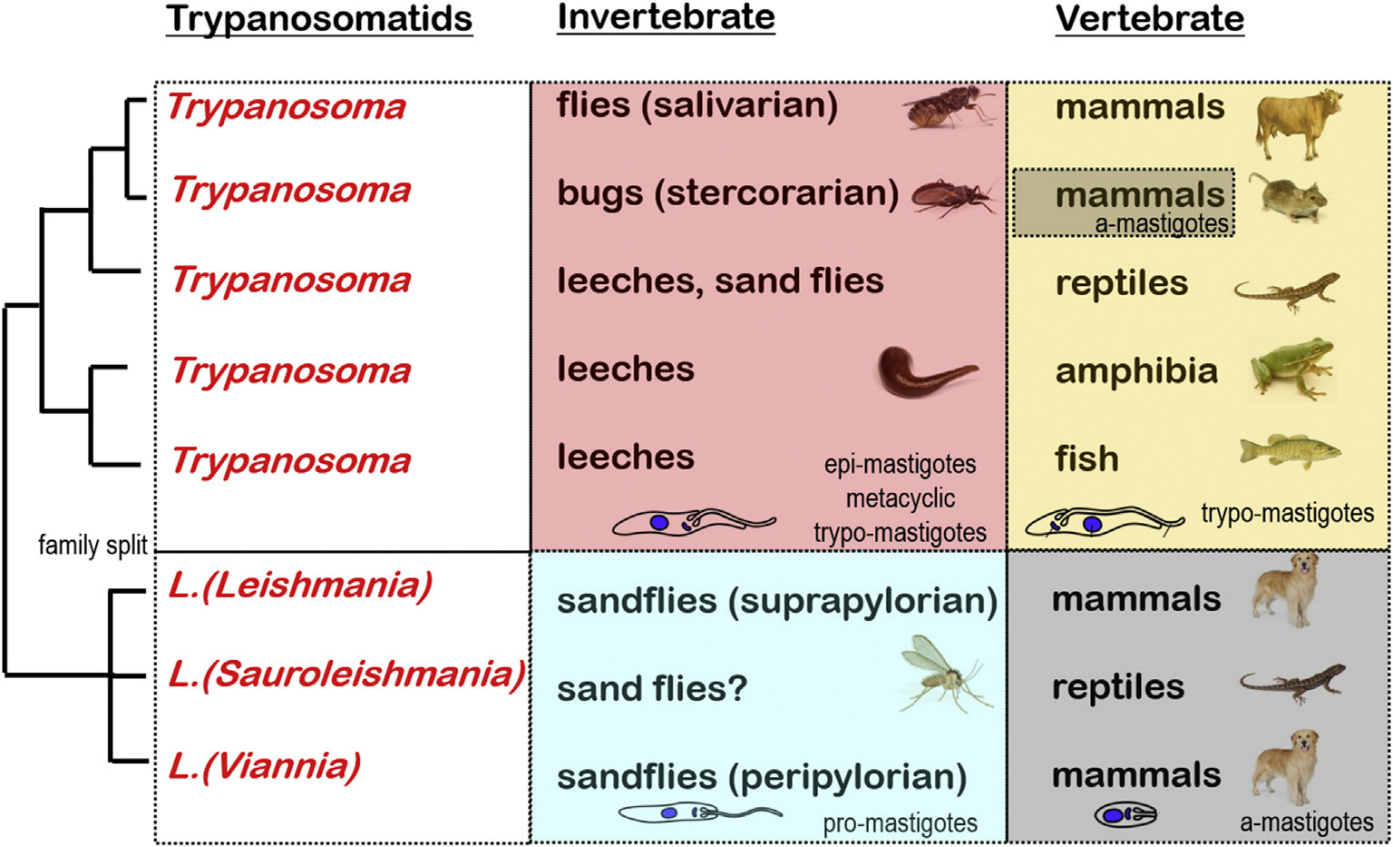
Phenotypic characters mapped against broad molecular phylogenies of trypanosomatid haemoflagellates. Molecular phylogenetic relationships are indicated on the left as a consensus (macro-evolutionary) tree derived from multiple studies cited within the text.

Studies on *hsp70* gene sequences of different *Leishmania* species by Fraga et al. (2010) revealed three clades which were referable to different subgenera: *L. Viannia* species causing New World cutaneous and mucocutaneous leishmaniasis and undergoing peri-pylorian (hindgut) development in *Lutzomyia* sandflies; *L. Leishmania* species causing New and Old World cutaneous and visceral leishmaniasis and undergoing supra-pylorian (foregut) development in *Phlebotomus* sandflies; and an intermediary *L. Sauroleishmania* group found in geckoes. Studies on trypanosomes of aquatic hosts using *ssurRNA* and *gGAPDH* gene sequences (cf. Hamilton et al., 2007, Hayes et al., 2014, Paparini et al., 2014) found marked differences between aquatic and terrestrial clades with additional clades for *Trypanosoma* species in freshwater fishes, marine fishes, amphibians, tortoises and platypuses: all thought to be transmitted by leech vectors. Collectively, molecular phylogenetic studies conducted on the dixenous trypanosomatid parasites have shown the family to be split into two major lineages (Fig. 4): one containing at least five clades of *Trypanosoma* species, and the other containing three clades of *Leishmania* species.

Certain distinct patterns emerge when mapping conventional phenotypic characters (involving parasite morphology and host occurrence) onto molecular phylogenies. First, all *Leishmania* groups form promastigote stages in the fore- or hind-guts of their vectors, which all happen to be sandflies. Second, all *Leishmania* species form amastigote stages in the tissues of their vertebrate hosts, which are mostly mammals (except for *L. Sauroleishmania* species which are transmitted to reptiles). Third, all *Trypanosoma* groups form epimastigote stages in their invertebrate vectors; those in flies and leeches undergoing anterior station (salivarian) development, and those in bugs undergoing posterior station (stercorarian) development. Fourth, all *Trypanosoma* species form trypomastigote stages in the blood of their vertebrate hosts (mammals, reptiles, amphibians, fish), with the notable exception of several stercorarian species that also form amastigote stages in host tissues (e.g. *T. cruzi*). In summary, contemporary molecular phylogenetic studies on blood-borne trypanosomes have largely reconciled well with conventional phenotypic groupings based on parasite developmental cycles. Robust phylogenetic comparisons with free-living kinetoplastid relatives suggest that parasitism arose several times during their evolution (Lukes et al., 2014), but not necessarily step-wise through particular host or vector assemblages nor sequentially through monoxenous then dixenous life-styles. Taking into account the vertebrate hosts and invertebrate vectors of these clades, it was concluded that ‘host-switching’ and/or ‘host-filling’, rather than co-speciation, were mechanisms involved in trypanosome evolution (Hamilton et al., 2007). Branch length analyses and molecular clock models suggest that parasitic trypanosomes arose first in aquatic invertebrates (deepest branches) and then radiated into terrestrial environments and vertebrates (Lukes et al., 2014). The placement of many species remains to be determined, especially those from aquatic environments with leech vectors.

## 5 Apicomplexa

The remaining four haemoprotozoan groups all belong to the phylum Apicomplexa: so-named because the trophic stages possess a unique complex of anterior (apical) organelles, including a conoid (spiral cone of microtubules), polar ring, micronemes (small electron-dense bodies), rhoptries (larger saccular electron-dense bodies), subpellicular microtubules and micropore, that facilitate host cell invasion for these intracellular parasites (with the exception of some extracellular stages for gregarines). All developmental stages have cell membranes supported by subpellicular alveoli, thereby placing them in the supergroup Alveolata together with the ciliates and dinoflagellates (Adl et al., 2012). Apicomplexan cells have elongate mitochondria and many have recently been found to possess a unique apicoplast organelle. Molecular phylogenies have been based on nuclear, mitochondrial and apicoplast DNA sequences (cf. Arisue and Hashimoto, 2015), while traditional classification systems have been based on parasite morphology and developmental cycles as well as host range and specificity (Levine, 1988). Blood-borne apicomplexan parasites are thought to have evolved first in invertebrate hosts and then in vertebrates. They are presumed to have free-living dinoflagellate-like ancestors that made the transition to parasitism when taken up by marine worms possibly by myzocytosis (cellular vampirism) to become intracellular parasites in which apical complex organelles developed. Comparative studies with other non-blood-borne apicomplexa (gregarines, coccidia) suggest they were then passed to arthropods (aquatic diptera and terrestrial beetles) and then to vertebrate hosts (via insect and non-insect vectors) (Garnham, 1966, Lee et al., 1985, Lee et al., 2000). Three major apicomplexan groups (gregarines, coccidia and haematozoa) are recognized primarily on the basis of host occurrence, tissue tropism and developmental cycle:

• Gregarines are found mainly in the gut or body cavity of marine worms and insects. They form large trophonts and gamonts which may be septate or aseptate, and all have an anterior holdfast organelle (epimerite or mucron) formed by a modified conoid. Gamonts recombine by isogamous fusion with syzygy.
• Coccidia form non-motile resistant spores (oocysts) containing infective sporozoites usually confined within secondary spores (sporocysts). These spores may be exogenous (being excreted into the external environment to contaminate food or water supplies) or endogenous (occurring in haematophagous invertebrates which transmit infections between hosts). Most species multiply asexually by fission or endogeny and undergo sexual reproduction by anisogamous fusion without syzygy. Unlike the gregarines, the gamonts are small, usually intracellular and lack a mucron or epimerite. Three groups of coccidia are recognized: coelotrophiid coccidia in marine annelids; adeleid coccidia in marine and terrestrial invertebrates (including blood parasites enigmatically known as ‘haemogregarines’ in reptiles and amphibians with leech or arthropod vectors); and eimeriid coccidia in vertebrates (including a few ‘haemococcidia’ in the blood of reptiles, amphibians and possibly birds).
• Haematozoa form stages with a reduced apical complex (lacking conoid) that parasitize blood cells and occasionally tissues of vertebrates and have haematophagous invertebrate vectors. Two main groups are recognized: haemosporidia which form pleomorphic ‘spores’ in mammals, birds and reptiles and are transmitted by dipteran vectors; and piroplasms which form pyriform ‘spores’ in mammals and are transmitted by tick vectors.

Over 4000 species of Apicomplexa have been described as parasites of vertebrate and invertebrate hosts. All undergo cyclic development involving up to three different divisional processes (Lee et al., 1985, Lee et al., 2000): asexual merogony (schizogony) either by fission (splitting of maternal cell) or endogeny (internal formation of daughter cells); gamogony involving formation of gametes (macrogametes = female, microgametes = male) which undergo fertilization to recombine by fusion (syngamy) with or without paired alignment (syzygy); and sporogony (formation of infective sporozoites). For apicomplexan blood parasites, vertebrate hosts often act as intermediate hosts only supporting asexual development of parasites, but with the formation of precursor/immature sexual stages (gamonts). While the genetics of recombination processes in parasites remain to be definitively determined, the formation of gamonts (supposedly haploid?) suggests that meiotic division of the apicomplexan haemoparasites commenced in the vertebrate hosts. However, gamete maturation and subsequent recombination (to diploidy?) through fertilization usually occurs in the invertebrate hosts, so by definition these vectors are considered to be definitive hosts.

**Fig. 5:**
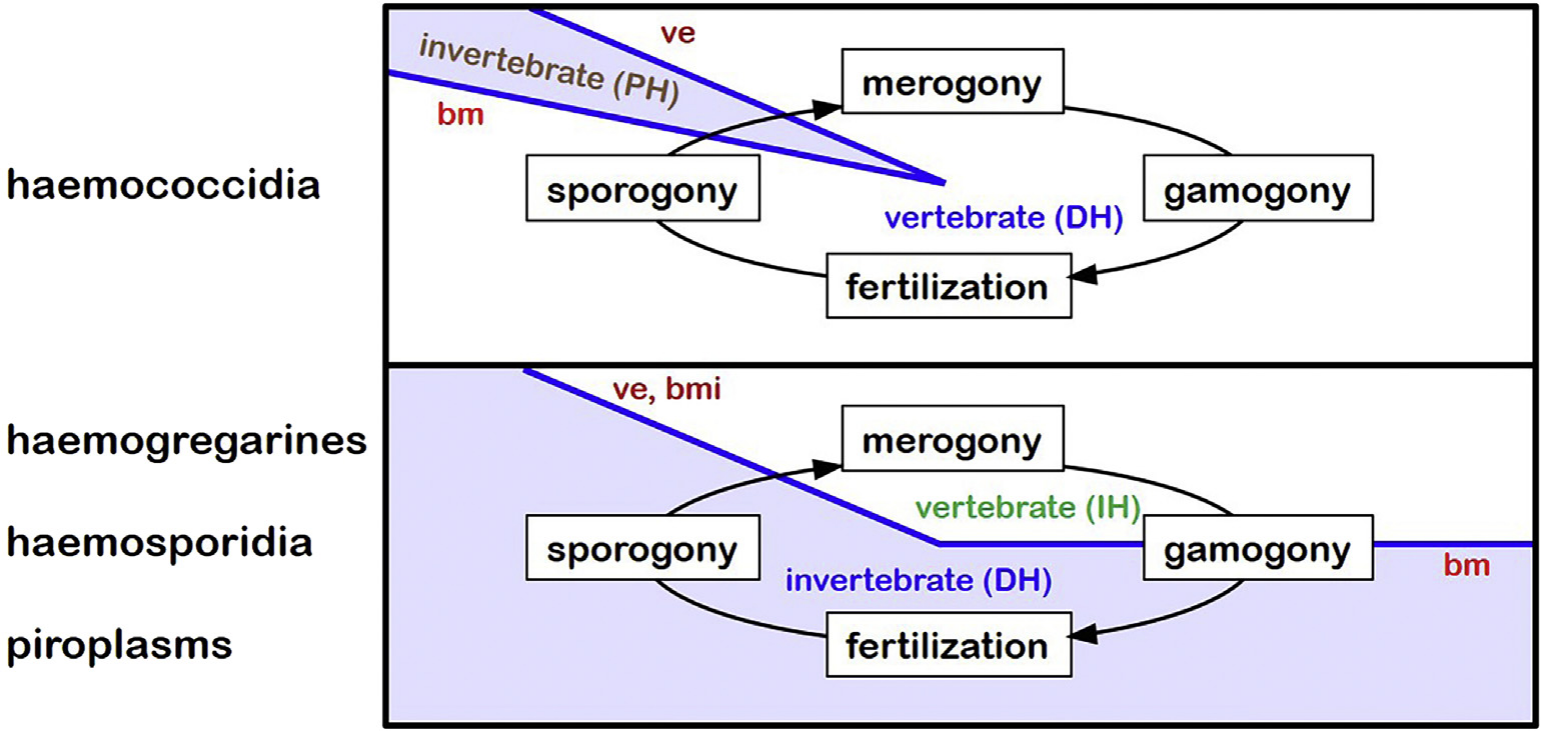
Developmental cycles and hosts for apicomplexan blood parasites (DH = definitive host; IH = intermediate host; PH = paratenic host; bm = blood meal; bmi = injected during blood meal; ve = vector eaten).

The developmental life-cycles of most blood-borne apicomplexans are remarkably similar, alternating between asexual development (merogony) in their vertebrate (intermediate) hosts and sexual development (gamogony and sporogony) in their invertebrate (definitive) hosts (Fig. 5). Haemogregarines, haemosporidia and piroplasms multiply in vertebrates and form gamont stages in host blood cells. These are taken up by blood-feeding invertebrates where they complete gamete development and fertilization producing zygotes which subsequently divide to form numerous infective sporozoites. Infections are transmitted to new vertebrate hosts when sporozoites are injected during vector feeding (for haemosporidia and piroplasms) or when infected vectors are ingested (eaten) by vertebrate hosts (for haemogregarines). In contrast, haemococcidia complete all of their development (merogony, gamogony and sporogony) in their vertebrate hosts (in this instance, acting as definitive hosts) and only use haematophagous invertebrates as paratenic hosts to transport sporozoites or merozoites between vertebrates (via insectivory when infected invertebrates are eaten by vertebrates).

On the basis of comparative biological characteristics, the haemococcidia are thought to have close affinities to other coccidians, particularly monoxenous gut coccidia rather than heteroxenous tissue cyst-forming coccidia. Haemogregarines are considered to be close cousins to the coccidia, while haemosporidia and piroplasms are thought to be more distantly related. It is interesting to note that modern molecular phylogenetic studies have tended to support relationships inferred from biological data, albeit at different discriminatory levels depending on the genetic sequences being compared. While analyses of partial gene sequences are excellent at detecting intra-specific variation (leading to the characterization of species, strains, isolates, genotypes, etc.), more broad-brush genomic studies are often required to help resolve higher taxonomic relationships (between genera, families, orders, classes, phyla, etc.). Such studies have supported the uniqueness (monophyly) of some of the different haemozoan assemblages as well as their broad evolutionary relationships (paraphyly) (cf. Arisue and Hashimoto, 2015). However, the situation is far from simple as the blood-borne apicomplexan groups are interspersed amongst the non-blood-borne groups (Morrison et al., 2004, Barta et al., 2012). The haemococcidia appear to be grouped within the enteric eucoccidia but quite distant from the adeleorine coccidia (and haemogregarines). The haemosporidia and piroplasms are well separated groups and both appear to have closer affinities to the adeleorine coccidia (and haemogregarines) than to the eucoccidia.

## 6 Haemococcidia

### 6.1 Biodiversity

Detailed analyses have been conducted on many different coccidian isolates harvested from host faeces, tissues or blood. All coccidia form oocysts (zygotes) in which infective sporozoites are produced (process of sporogony). Conventional eucoccidian groups include the enteric coccidia (most occurring as monoxenous parasites of vertebrates with faecal-oral transmission) and the tissue cyst-forming coccidia (most occurring as heteroxenous parasites of vertebrates with predator-prey transmission cycles). The haemococcidia are quite different from the other groups in that they complete development in their vertebrate hosts and only use invertebrates as paratenic transport hosts.

For those eimeriid coccidia known to have a blood-borne stage (i.e. the haemococcidia), all asexual and sexual division occurs in the tissues of vertebrate hosts (reptiles, amphibians and birds) but some zoites (sporozoites or merozoites) end up in blood cells where they may be taken up by blood-feeding invertebrates (leeches, arthropods). These stages do not undergo further development but are infectious to other vertebrates when they eat infected invertebrates. Haemococcidia have been described from two eimeriid families; *Lankesterella*, *Lainsonia* and *Schellackia* from the family Lankesterillidae, and *Atoxoplasma* (now considered to be *Isospora* species) from the family Eimeriidae (Table 2). All species undergo merogony in the intestines or reticulo-endothelial (RE) cells of their vertebrate hosts and some zoites end up in blood cells (sporozoites in the case of *Lankesterella*, *Lainsonia* and *Schellackia*, and merozoites in the case of *Atoxoplasma* (*Isospora*) species). In many instances, these infected blood cells are thought to be ingested by invertebrate vectors which are subsequently eaten by other vertebrates as part of their food cycles.

**Table 2:** Genera of haemococcidian parasites.

Assemblage	Genus	No. spp.	Site of development in vertebrate		Vertebrate hosts	Invertebrate vectors
			meronts	gamonts		
F: Lankesterellidae	*Lankesterella*	9	RE cells	RE cells	lizards, frogs	leeches, arthropods
F: Lankesterellidae	*Lainsonia*	2	RE cells	RE cells	lizards	mosquitoes
F: Lankesterellidae	*Schellackia*	13	intestines	RE cells	lizards, snakes, frogs	mites, mosquitoes
F: Eimeriidae	*Atoxoplasma* (*Isospora* p.p.)	5	intestines	blood cells	birds	none

### 6.2 Molecular clades

**Fig. 6:**
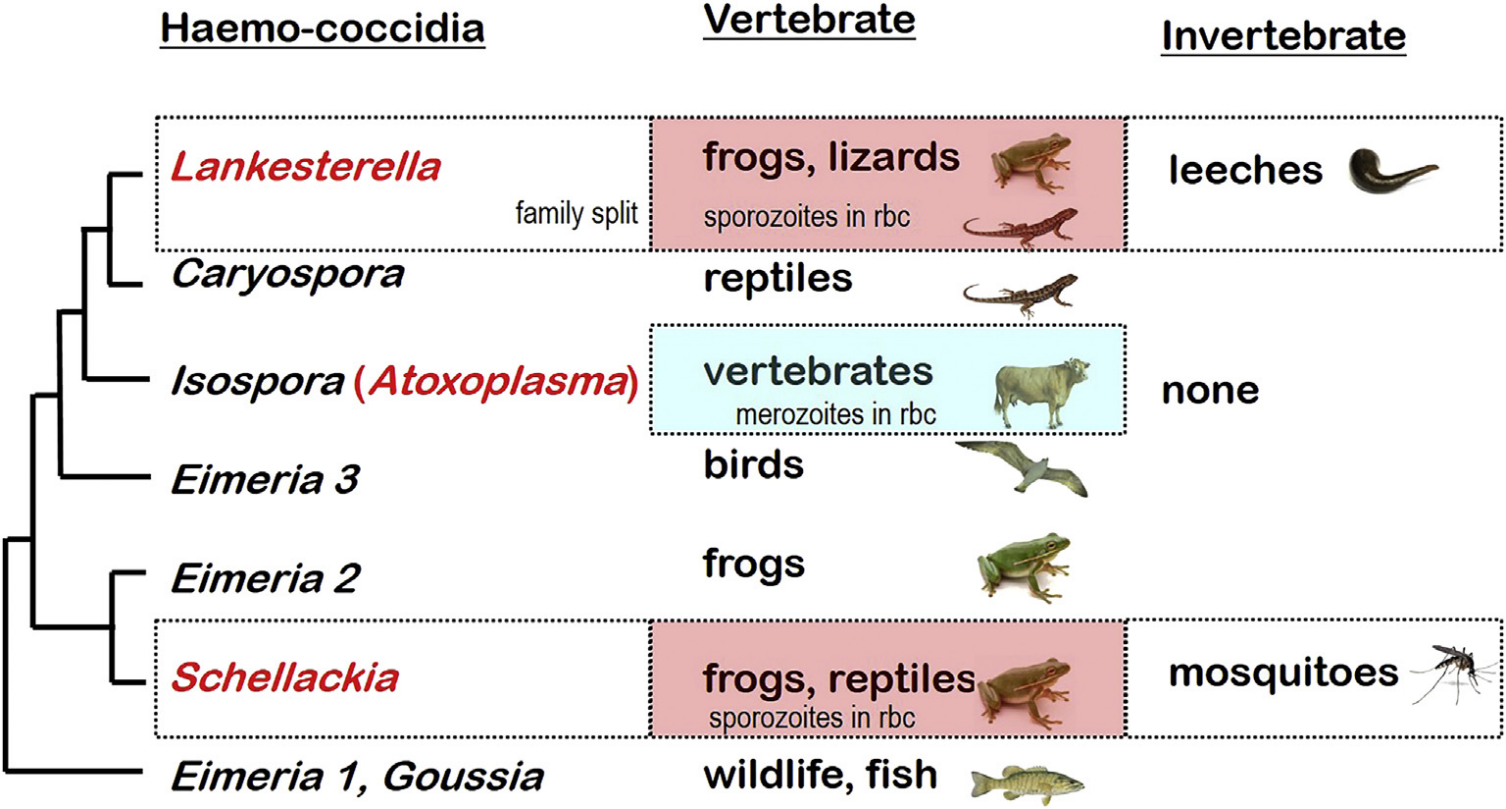
Phenotypic characters mapped against broad molecular phylogenies of haemococcidian parasites (blood-borne genera shown in red). Molecular phylogenetic relationships are indicated on the left as a consensus (macro-evolutionary) tree derived from multiple studies cited within the text. (For interpretation of the references to colour in this figure legend, the reader is referred to the web version of this article.)

Molecular studies on *ssurRNA* gene sequences have long shown good separation between the monoxenous enteric coccidia and the heteroxenous tissue cyst-forming coccidia (cf. Morrison et al., 2004, Barta et al., 2012), with some studies suggesting the haemococcidia may be an intermediary group. However, more recent comprehensive studies (cf. Merino et al., 2006, Megia-Palma et al., 2013, Megia-Palma et al., 2014) have shown that *Lankesterella* and *Schellackia* do not group together but are interspersed amongst the monoxenous enteric coccidia. Similarly, *Atoxoplasma* was not grouped with other haemococcidia but was nested with some avian *Isospora* species amongst the monoxenous enteric coccidia (cf. Schrenzel et al., 2005). Taking into account these collective results, the haemococcidia are not considered a natural assemblage of organisms. Instead, they are split between several independent groups - the majority of which comprise enteric coccidia within vertebrates (Fig. 6).

*Atoxoplasma* species group together with many *Eimeria* and *Isospora* species from vertebrates. While *Atoxoplasma* merozoites have been observed in blood cells leading to the continuance and dissemination of infection within individual hosts, they have not been shown to be vector-borne or involved in horizontal transmission. In contrast, *Lankesterella* species in frogs and lizards group most closely with *Caryospora* species in other reptiles, while *Schellackia* species in frogs and reptiles groups together with *Eimeria* species in frogs. Both *Lankesterella* and *Schellackia* species form sporozoites in host blood cells which are taken up by invertebrate paratenic hosts (leeches and mosquitoes respectively). These haemococcidian species are thought to have evolved in vertebrates from gut coccidia and only recently involved invertebrates as paratenic hosts. Indeed, several other coccidian species belonging to the genera *Caryopsora* and *Cystoisospora* have recently been shown to involve paratenic transport hosts in their life-cycles. Further studies are required to investigate the host ranges and specificities of these coccidian parasites.

## 7 Haemogregarines

### 7.1 Biodiversity

In contrast to the haemococcidia which only use invertebrates as paratenic transport hosts, the haemogregarines do have obligative heteroxenous life cycles involving invertebrate vectors in which parasite development must take place before transmission to vertebrate hosts. Haemogregarines are classified together with other coccidia, specifically with the adeleorine coccidia in marine and terrestrial animals. These coccidia undergo cyclic merogony and gamogony in vertebrates but fertilization and sporogony occurs in invertebrate vectors (Lee et al., 1985, Lee et al., 2000). The macro- and micro-gamonts are associated pairwise in syzygy and therefore few microgametes are produced (usually 1–4). The sporozoites are usually enclosed in secondary envelopes (i.e. sporocysts) which are usually transmitted to new vertebrate hosts when the vectors are eaten. Some species do not produce characteristic sporocysts and some vectors infect their vertebrate hosts by inoculative transmission. Around 400 haemogregarine species have been recorded as heteroxenous parasites mainly in vertebrate blood cells and various blood-sucking invertebrate vectors.

Haemogregarines are common in reptiles and some occur in fish, amphibians, birds and mammals. Most studies have been confined to intra-erythrocytic life-cycle stages (almost exclusively gamonts) which are readily detected in blood smears. Numerous species were described simply on the basis of host occurrence but little is known about their actual host specificity or developmental cycles. Despite the early confusion, there has been growing acceptance of the recent classification of haemogregarines into four families (Lee et al., 2000) (Table 3): Haemogregarinidae containing the genera *Haemogregarina*, *Cyrilia* and *Desseria*; Hepatozoidae containing the genus *Hepatozoon;* Karyolysidae containing the genera *Karyolysus* and *Hemolivia*; and Dactylosomatidae containing the genera *Dactylosoma* and *Babesiosoma*. It has been suggested that the chelonian (tortoise) haemogregarines be regarded as *Haemogregarina* sensu stricto (in the strictest sense) whereas fish haemogregarines be classified as *Desseria* or *Cyrilia* or left as *Haemogregarina* sensu lato (in the broadest sense) and that all members of the genus *Haemogregarina* in snakes, crocodiles, lizards, amphibians, birds and mammals be transferred to the genus *Hepatozoon* pending determination of their vectors and studies on their developmental cycles (Siddall, 1995). All haemogregarines use vertebrates (mammals, reptiles, amphibians, birds or fish) as intermediate hosts for cyclic merogony (often forming macro-then micro-merozoites) in tissues and gamont development in blood cells (mostly red, sometimes white, blood cells). Gametogony is completed in invertebrate definitive hosts (leeches, acarines or dipterans) followed by fertilization and oocyst formation in the gut or haemocoel. The oocysts undergo sporogony resulting in sporozoites lying directly within oocysts (for Dactylosomatidae), within sporocysts (for Hepatozoidae), within tissues (for Haemogregarinidae) or within eggs (for Karyolysidae).

**Table 3:** Genera of haemogregarine parasites.

Assemblage	Genus	No. spp.	Site of development in vertebrate		Vertebrate hosts	Invertebrate vectors
			meronts	gamonts		
F: Haemogregarinidae	*Cyrilia*	4	erythrocytes	erythrocytes	fish	leeches
F: Haemogregarinidae	*Desseria*	40	erythrocytes	erythrocytes	fish	leeches
F: Haemogregarinidae	*Haemogregarina*	46	erythrocytes	erythrocytes	turtles	leeches
F: Hepatozoidae	*Hepatozoon*	300	viscera	erythrocytes, leucocytes	mammals, birds, reptiles, amphibia, fish	leeches, arthropods
F: Karyolysidae	*Hemolivia*	4	RE cells	erythrocytes	lizards, tortoises, toads	ticks
F: Karyolysidae	*Karyolysus*	5	viscera ± rbc	erythrocytes	lizards	mites
F: Dactylosomatidae	*Dactylosoma*	10	erythrocytes	erythrocytes	chameleons, frogs, teleosts	leeches
F: Dactylosomatidae	*Babesiosoma*	7	erythrocytes	erythrocytes	frogs, fish	leeches

Early phylogenetic studies used cladistics analyses of multiple phenotypic characters (morphology, biology) to reveal several clades apparently associated with different vectors (notably ticks, flies and leeches) rather than particular vertebrate hosts (cf. Siddall, 1995, Smith and Desser, 1997, Smith et al., 2000, Jakes et al., 2001), thus suggesting vector-first evolution. However, such patterns may have arisen due to the environments in which the hosts occurred; e.g. marine hosts with leeches, and terrestrial hosts with ticks or flies.

### 7.2 Molecular clades

Molecular phylogenetic studies using *ssurRNA* gene sequences revealed any associations between haemogregarines and their vertebrate hosts to be paraphyletic with many mixed clades associated with parasites in snakes, lizards, amphibians and mammals (cf. Wicks et al., 2006, Cook et al., 2014, Haklova-Kocikova et al., 2014). Detailed analyses revealed five major clades (Kvicerova et al., 2014): two *Hepatozoon* clades (clade A associated with carnivores and clade C with reptiles and frogs) separated by an intermediary *Hemolivia* clade in lizards, and two distant clades, one containing *Haemogregarina* species from tortoises and the other containing *Dactylosoma/Babesiosoma* species from fish and frogs. The five major clades were also well separated with respect to vectors: the former three clades using ticks and flies as vectors and the latter two using leeches.

**Fig. 7:**
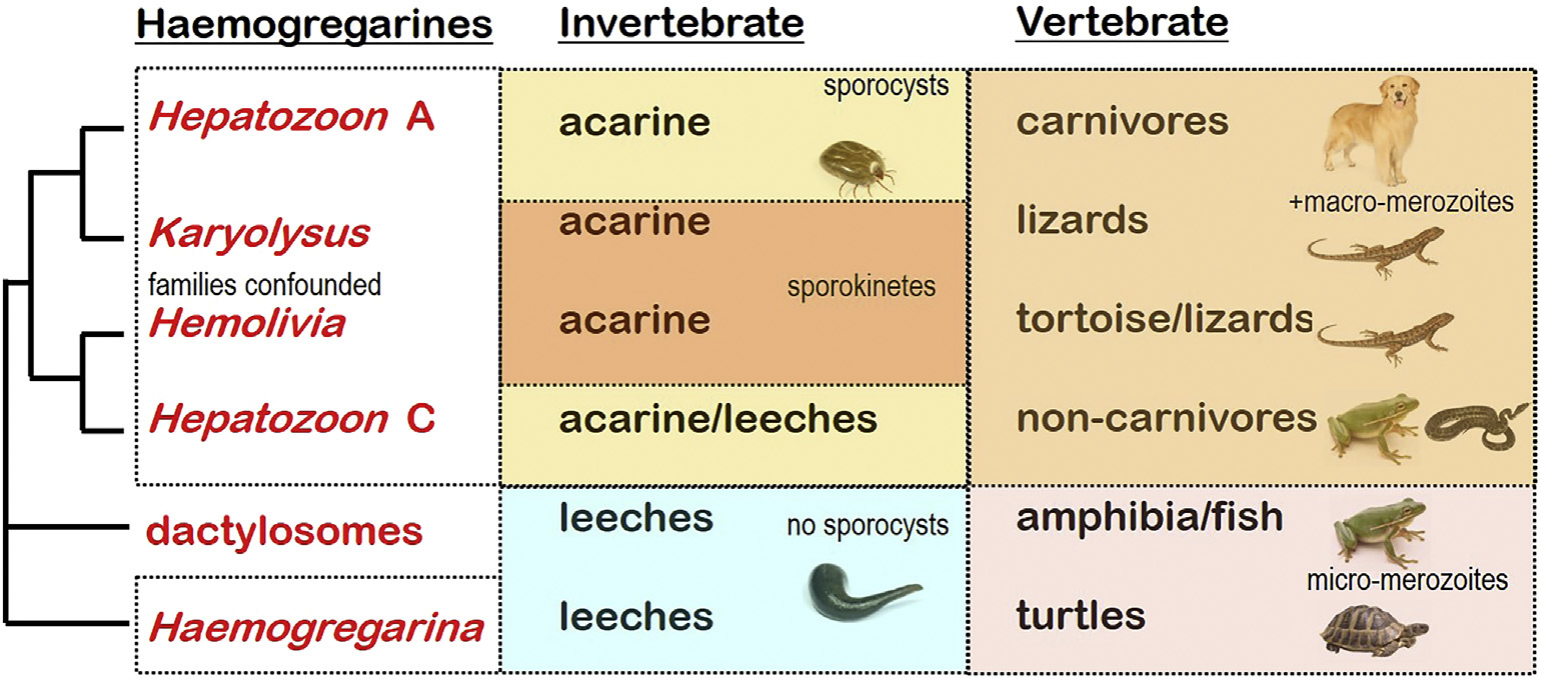
Phenotypic characters mapped against broad molecular phylogenies of haemogregarine parasites. Molecular phylogenetic relationships are indicated on the left as a consensus (macro-evolutionary) tree derived from multiple studies cited within the text.

Mapping biological characters onto molecular phylogenies revealed many of the conventional families to be confounded (Fig. 7), e.g. the *Hepatozoon* species in mammals (clade A) were more closely related to *Karyolysus* species from lizards, while the *Hepatozoon* species in frogs and reptiles (clade C) were more closely related to *Hemolivia* species from tortoises and lizards. Nonetheless, several interesting biological associations were evident between paraphyletic clades. *Haemogregarina* sensu stricto and dactylosome clades use leech vectors without sporocyst formation, and they only formed micro-merozoites in their turtle, frog or fish hosts. In contrast, members of the *Hepatozoon*, *Karyolysus* and *Hemolivia* clades use tick vectors with sporocyst (or sporokinete) formation, and they formed macro-merozoites in their mammal or reptile hosts. It is currently thought that haemogregarines evolved first in invertebrates (along with many other adeleorine coccidia and gregarines) but then diverged once they incorporated vertebrates into their life-cycles. It will be interesting to test the two major transitions in haemogregarine life-styles: firstly the transition from leeches to arthropods, and secondly, the transition from aquatic to terrestrial environments and hosts.

## 8 Haemosporidia

### 8.1 Biodiversity

While most haemogregarines develop in invertebrate vectors and are transmitted to vertebrates by predation (insectivory or ingestion of infected vectors), haematozoan parasites develop in invertebrates and are injected into vertebrates during blood-feeding (inoculative transmission). Two main haematozoan groups are recognized: haemosporidia which form pleomorphic ‘spores’ in mammals, birds and reptiles and are transmitted by dipteran vectors; and piroplasms which form pyriform ‘spores’ in mammals and are transmitted by tick vectors (Lee et al., 1985, Lee et al., 2000). Haemosporidian parasites have a reduced apical complex (lacking conoid) and their sexual multiplication does not involve syzygy (gametes develop independently). Following fertilization in the invertebrate vector, the resultant zygote (ookinete) becomes motile and moves through the gut wall and undergoes sporogony without sporocyst formation in vector tissues, notably the salivary glands.

Over 540 species belonging to some 20 haemosporidian genera have been described from a wide range of mammalian, avian and reptilian hosts around the world (Garnham, 1966, Levine, 1988) (Table 4); most causing little harm to their hosts but some causing devastating diseases (including malaria in humans). Four families are recognized mainly on the basis of their developmental cycles and whether haemozoin pigment is produced as a by-product of haemoglobin digestion by the parasites (cf. Valkiunas, 2005): namely, Plasmodiidae (merogony in tissues then cyclic in erythrocytes, pigment present); Haemoproteidae (merogony in tissues only, pigment present); Leucocytozoidae (merogony in tissues, pigment absent) and Garniidae (merogony in leucocytes, pigment absent). Vertebrates act as intermediate hosts in which the parasites undergo asexual multiplication either within tissues or blood cells or both.

**Table 4:** Genera of haemosporidian parasites (* = haemozoin present).

Assemblage	Genus	No. spp.	Site of development in vertebrate		Vertebrate hosts	Invertebrate vectors
			meronts	gamonts		
F: Plasmodiidae	*Plasmodium*	200	liver, blood	erythrocytes*	mammals, birds, reptiles	diptera
F: Plasmodiidae	*Hepatocystis*	25	liver	erythrocytes*	primates/bats	midges
F: Plasmodiidae	*Polychromophilus*	6	viscera	erythrocytes*	bats	nycterids
F: Plasmodiidae	*Nycteria*	7	liver	erythrocytes*	bats	?
F: Plasmodiidae	*Biguetiella*	1	liver	erythrocytes*	bats	?
F: Plasmodiidae	*Bioccala*	1	RE cells	erythrocytes*	bats	?
F: Plasmodiidae	*Dionisia*	1	liver	erythrocytes*	bats	?
F: Plasmodiidae	*Rayella*	3	liver	erythrocytes*	flying squirrels	?
F: Plasmodiidae	*Billbraya*	1	erythrocytes	erythrocytes*	lizards	?
F: Plasmodiidae	*Mesnilium*	1	RE cells	blood cells*	fish	leeches
F: Plasmodiidae	*Haemocystidium*	6	RE cells	erythrocytes*	lizards/tortoises	arthropods
F: Haemoproteidae	*Haemoproteus (Halteridium)*	20	RE cells	erythrocytes*	birds	louse flies
F: Haemoproteidae	*Parahaemoproteus*	150	RE cells	erythrocytes*	birds	midges
F: Haemoproteidae	*Johnsprentia*	1	RE cells	erythrocytes*	flying foxes	?
F: Haemoproteidae	*Simondia*	1	RE cells	erythrocytes*	turtles	insects
F: Haemoproteidae	*Sprattiella*	1	RE cells	erythrocytes*	bats	?
F: Leucocytozoidae	*Leucocytozoon (Akiba)*	100	RE cells	blood cells	birds	blackflies
F: Leucocytozoidae	*Saurocytozoon*	2	viscera	leucocytes	lizards	mosquitoes
F: Garniidae	*Garnia*	7	leucocytes	leucocytes	lizards	arthropods
F: Garniidae	*Progarnia*	1	blood cells	blood cells	crocodiles	?
F: Garniidae	*Fallisia*	8	leucocytes	leucocytes	lizards	arthropods

In vertebrates, all haemosporidian parasites form gamonts in host blood cells after they have undergone asexual merogony either in tissues (for families Haemoproteidae and Leucocytozoidae), in tissues then in erythrocytes (for family Plasmodiidae) or in leucocytes (for family Garniidae) (Valkiunas, 2005). Haemozoin pigment is only formed by members of two families (Plasmodiidae and Haemoproteidae). Parasites may cause disease in vertebrates either by merogony within tissues causing lesions or by merogony within erythrocytes causing ‘malaria’ characterized by cyclic fevers, haemolytic anaemia (exacerbated by erythrophagocytosis) and ischaemia (due to cytoadherence, rosetting, disseminated intravascular coagulation and haemozoin deposition). Gamonts in blood cells taken up by dipteran vectors may complete their development and undergo fertilization in the gut forming a motile zygote (ookinete) which penetrates the gut and forms oocysts in tissues. The oocysts undergo sporogony and produce thousands of sporozoites which are injected into vertebrate hosts when the vectors feed.

### 8.2 Molecular clades

Phylogenetic studies on haemosporidian parasites have concentrated on *Plasmodium* species causing malaria in humans in attempts to identify their evolutionary origins and relationships. Consensus trees formulated from studies on nuclear, mitochondrial and apicoplast genes have grouped *Plasmodium* species from primates separately to those from other mammals, birds and reptiles (cf. Keeling and Rayner, 2015). *Plasmodium* species infecting humans were not monophyletic, but occurred in four separate clades involving other primates. Previous studies demonstrated that *P. falciparum* had close affinities to parasites from Africa apes while *P. vivax* and *P. malariae* were more closely related to isolates from New World monkeys (cf. Carter and Mendis, 2002), but recent studies have indicated that all *Plasmodium* species infecting humans may have originated from African apes (gorillas and chimpanzees) with subsequent radiation of some to New World monkeys (Loy et al., 2017).

Many studies have shown that while mammalian *Plasmodium* species group together (broadly), those species infecting birds and reptiles fell into several paraphyletic clades (cf. Perkins and Schall, 2002, Yotoko and Elisei, 2006, Outlaw and Ricklefs, 2010, Outlaw and Ricklefs, 2014). Not only were these clades well separated in evolutionary time, they were mixed in that some bird and lizard isolates were more closely related to each other than other bird and lizard isolates (cf. Pineda-Catalan et al., 2013). When other haemosporidian genera were included in analyses (cf. Witsenburg et al., 2012), it was found that genera from bats were paraphyletic - *Hepatocystis* isolates grouped together with mammalian *Plasmodium* species (cf. Thurber et al., 2013), while *Polychromophilus* isolates grouped separately with some reptile *Plasmodium* species. *Haemoproteus* and *Leucocytozoon* species were pleisiomorphic and grouped separately in individual clades (cf. Martinsen et al., 2006, Javanbakht et al., 2015). Further studies (e.g. concatenated studies on *ef2, asl, clpC* and *ctyb* sequences by Schaer et al., 2015) confirmed the duality of bat haemosporidia (*Hepatocystis* with close affinities to mammalian *Plasmodium,* but *Nycteria* and *Polychromophilus* more closely related to avian *Plasmodium*). They also reaffirmed the earlier ancestral relationships of the avian haemosporidia (*Haemoproteus*, *Parahaemoproteus* and *Leucocytozoon*) (cf. Balasubramaniam et al., 2013).

A seminal study by Martinsen et al. (2008) on consensus nuclear, mitochondrial and apicoplast gene sequences demonstrated a clear relationship between haemosporidian genera not only with their vertebrate hosts but also their invertebrate vectors. There were clear groupings of *Leucocytozoon* from birds, *Haemoproteus* from birds, *Parahaemoproteus* from birds, *Plasmodium* from birds and reptiles, *Plasmodium* from rodents and primates, and *Hepatocystis* from bats. These groups were clearly associated with different vectors; namely, simuliids (black-flies), hippoboscids (louse-flies), ceratopogonids (midges), culicine mosquitoes, anopheline mosquitoes, and midges, respectively. A growing number of studies are also comparing host and parasite phylogenetic trees (tanglegrams) to demonstrate varying degrees of co-evolution (cf. Santiago-Alarcon et al., 2014).

**Fig. 8:**
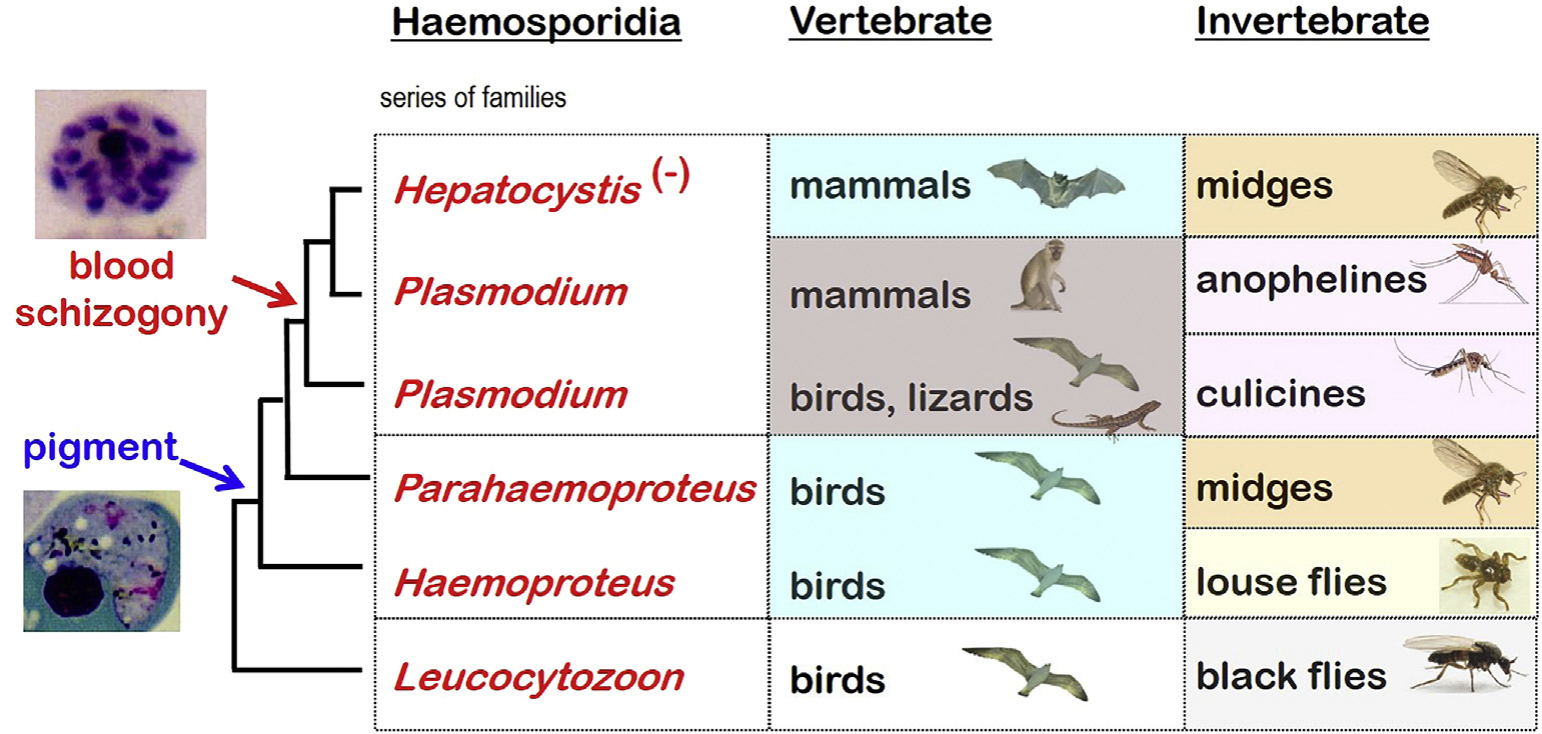
Phenotypic characters mapped against broad molecular phylogenies of haemosporidian parasites. Molecular phylogenetic relationships are indicated on the left as a consensus (macro-evolutionary) tree derived from multiple studies cited within the text.

When mapping phenotypic characters onto these molecular phylogenetic trees, several clear distinctions become apparent between and within haemosporidian genera (Fig. 8). There appears to have been a transition from haemosporidia which do not form haemozoin pigment (*Leucocytozoon* in white blood cells) to genera that do form pigment indicating haemoglobin digestion by parasites in red blood cells (*Haemoproteus*, *Parahaemoproteus*, *Plasmodium* and *Hepatocystis*). This was followed by a transition from haemosporidia which undergo schizogony exclusively in host tissues (*Leucocytozoon*, *Haemoproteus* and *Parahaemoproteus*) to those that undergo schizogony in blood cells (*Plasmodium*). While there appears to be a general shift in haemosporidian genera from birds and reptiles to mammals (from nucleated to non-nucleated blood cells), each genus is associated with a particular vector group: black-flies transmitting *Leucocytozoon* to birds; louse-flies transmitting *Haemoproteus* to birds; midges transmitting *Parahaemoproteus* to birds; culicine mosquitoes transmitting *Plasmodium* to birds and lizards; and anopheline mosquitoes transmitting *Plasmodium* to mammals. The exception to this general trend was *Hepatocystis* which does not undergo blood schizogony and is transmitted to bats by midges. Further studies on vector behaviours and host-specificity may enable us to envisage haemosporidian evolution from a historical zoogeographic or ecological perspective. However, there are another 15 haemosporidian genera that need to be spliced into this story (cf. Table 4), particularly those from bats (cf. Landau et al., 2012). The closest relatives of the haemosporidia are the eucoccidia which are common in vertebrate hosts (while gregarines are common in invertebrates). At present, evidence suggests that the haemosporidia evolved from coccidia in vertebrates, and then involved invertebrates in their transmission. This is all the more remarkable because the new invertebrate hosts became the definitive hosts in which sexual reproduction of the parasite was completed, rather than being intermediate or paratenic hosts supporting asexual stages. The events associated with haemosporidian parasites switching hosts (both vertebrate and invertebrate) are not known, but hopefully more comprehensive molecular clock studies (Bensch et al., 2013) may allow the chronology of these events to be estimated and compared to the evolutionary time frames of the hosts themselves.

## 9 Piroplasms

### 9.1 Biodiversity

Conventionally, the sister group to the haemosporidia is another apicomplexan group known as the piroplasms, so-named because they form small pear-shaped stages in vertebrate blood cells. These stages are taken up by haematophagous arachnids (ticks) where they multiply and are injected into new vertebrate hosts (inoculative transmission). Like the haemosporidia, piroplasms form stages in which the apical complex is reduced (conoid absent). Unlike the haemosporidia, piroplasms do not form haemozoin pigment, they form pyriform (rather than pleomorphic) ‘spores’ in mammalian blood cells, and they are transmitted by tick (rather than dipteran) vectors (Lee et al., 1985, Lee et al., 2000). Piroplasms undergo asexual reproduction by binary fission and schizogony in vertebrates and ticks, and recent studies have shown they may also undergo a unique process of sexual reproduction in ticks. Over 130 piroplasm species have been described in mammals and some birds, reptiles and fish. They have been classified into three families primarily on their site of development within their vertebrate hosts (Table 5): Babesiidae developing only in erythrocytes in mammals and some birds; Theileridae developing firstly in lymphocytes then erythrocytes in mammals; and Haemohormidiidae developing in nucleated erythrocytes in reptiles and fish.

**Table 5:** Genera of piroplasm blood parasites.

Assemblage	Genus	No. spp.	Site of development in vertebrate		Vertebrate hosts	Invertebrate vectors
			meronts	gamonts		
F: Babesiidae	*Babesia*	100	erythrocytes	erythrocytes	mammals, birds	ticks
F: Theileridae	*Theileria*	15	lymphocytes	erythrocytes	mainly ruminants,	ticks
F: Theileridae	*Cytauxzoon*	4	vascular endothelia	erythrocytes	carnivores	ticks
F: Haemohormidiidae	*Sauroplasma* *(Serpentoplasma)*	3	erythrocytes	erythrocytes	lizards, snakes	ticks?
F: Haemohormidiidae	*Haemohormidium* *(Haematractidium)*	12	erythrocytes	erythrocytes	tortoises, frogs, fish	leeches

Most species use ticks as vectors, including 1-, 2- and 3-host tick species. To maintain infections in ticks throughout their life-cycles, all piroplasms must be able to undergo horizontal (trans-stadial) transmission between different tick life-cycle stages (larvae, nymphs, adults). Some babesiid species also exhibit vertical (trans-ovarian) transmission from adult female ticks to their eggs (especially important for the transmission of piroplasms by 1-host ticks). In contrast, *Haemohormidium* species are thought to use aquatic leeches as vectors for their transmission to tortoises, frogs and fish.

Piroplasms multiply in vertebrate hosts by asexual cyclic merogony (budding, fission) in host blood cells, often resulting in disease typified by anaemia, jaundice and fever (syndromes known as tick fevers). After ticks have fed on infected host blood, some parasites are liberated from ingested erythrocytes in the tick gut and form elongate stages (gametocytes?) with unique arrowhead-shaped orgenelles (ray-bodies or Strahlenkorper). These bodies pair up (syzygy) and fuse (syngamous fertilization?) to produce a nonmotile zygote which uses its spike-like arrowhead organelle upon contact to invade an epithelial cell and transform into a motile ookinete stage. These stages penetrate the gut wall into the body cavity and invade tick tissues where they form more kinetes (sporokinetes). These stages undergo trans-stadial transmission, thus infections persist during metamorphosis from larvae to nymphs to adults. Kinetes of *Babesia* species may also invade the tick ovary and penetrate developing eggs where they divide to form small round organisms. These stages facilitate trans-ovarian transmission, so that nearly all tick progeny are born already infected. Some kinetes ultimately invade the salivary glands and form polymorphic multinucleate sporonts (or sporoblasts) which produce hundreds of vermiform sporozoites through a budding process. When the tick next feeds, the sporozoites are injected with tick saliva into the vertebrate host.

### 9.2 Molecular clades

Molecular phylogenetic studies using *ssurRNA* gene sequences (cf. Hunfeld et al., 2008, Sivakumar et al., 2014) clearly distinguished between theileriid and babesiid piroplasms, and further identified several clades for the latter, namely unguli-babesids (e.g. *B. bovis*), babesiids (e.g. *B. canis*), proto-theilerids (e.g. *B. gibsoni*) and archaeo-piroplasmids (e.g. *B. microti*). To date, six broad clades have been identified: I. ‘*Babesia microti’* clade comprising species from rodents; II. ‘Western’ clade from deer and dogs in the United States of America; III. *Theileria/Cytauxzoon* clade from felids; IV. *Theileria* clade from equids and rhinocercoses; V. *Theileria* clade from bovids; and VI. *Babesia* clade from ruminants, carnivores and rodents (cf. Schnittger et al., 2012, Leclaire et al., 2015). Collectively, molecular studies have shown that the genus *Babesia* may be split into two major lineages (*Babesia* sensu stricto and *Babesia* sensu lato) apparently split by a third intermediary lineage (*Theileria/Cytauxzoon*).

**Fig. 9:**
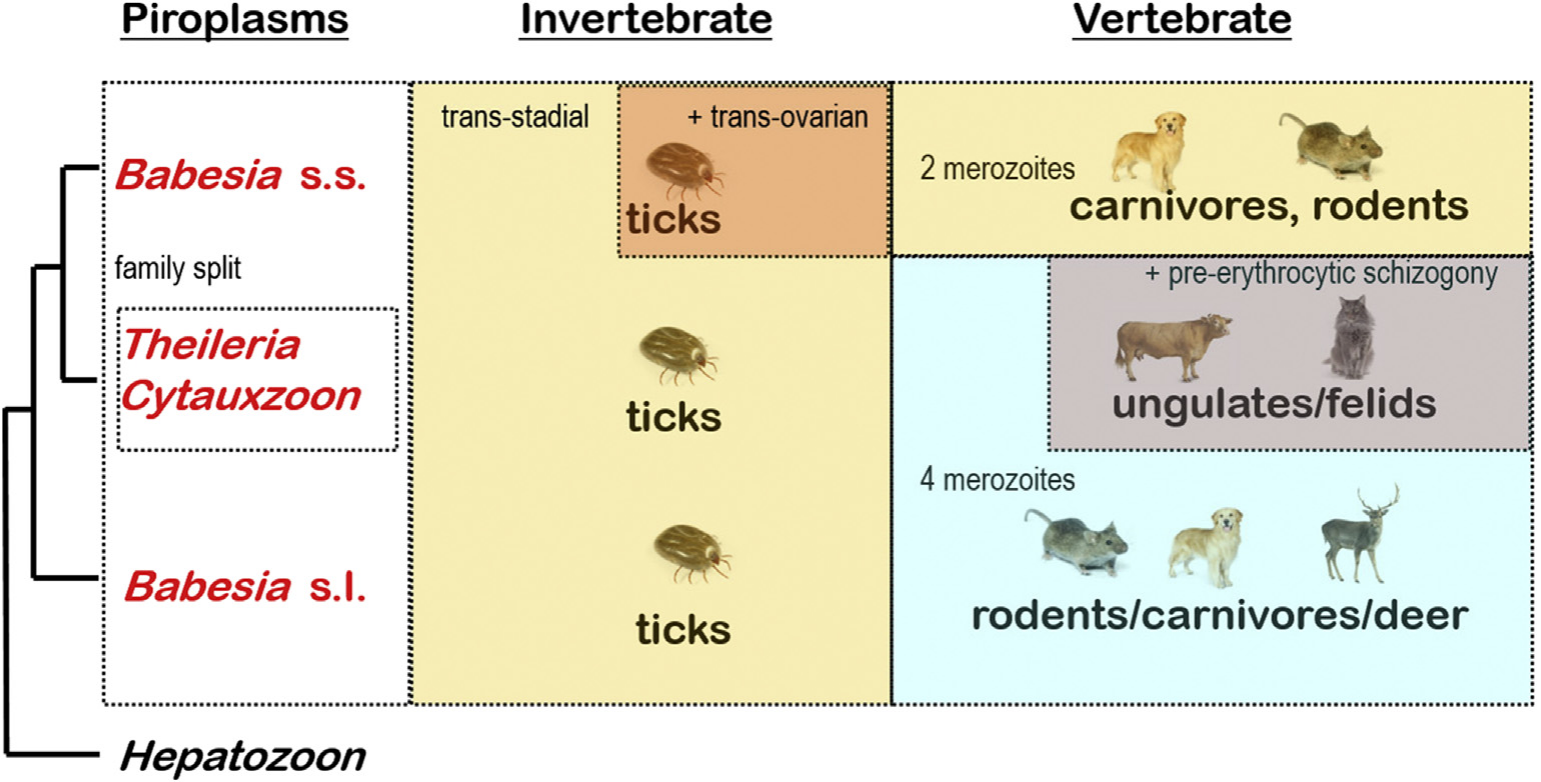
Phenotypic characters mapped against broad molecular phylogenies of piroplasm blood parasites. Molecular phylogenetic relationships are indicated on the left as a consensus (macro-evolutionary) tree derived from multiple studies cited within the text.

When mapping biological characteristics onto these broad phylogenetic divides, several features emerged (Fig. 9). The *Babesia* sensu stricto clade comprised those species which formed two merozoites in the erythrocytes of ruminants, carnivores and rodents, while the *Babesia* sensu lato clade consisted of those species which formed four merozoites in the erythrocytes of rodents, carnivores and deer. Similarly, species belonging to the intermediary *Theileria/Cytauxzoon* clade formed four merozoites in the erythrocytes of ungulates and felids, but they also exhibited pre-erythrocytic schizogony (which does not occur for any *Babesia* species). Piroplasms in all clades exhibited trans-stadial transmission in their tick vectors, but only those of the *Babesia* sensu stricto clade underwent trans-ovarian transmission.

Curiously, the piroplasms were found to be more closely related to some haemogregarines rather than to the haemosporidia, thus suggesting a common ancestry with the more ‘primitive’ adeleorine coccidia rather than with the eucoccidia. Evolutionary theories about the origins of piroplasms are confounded by the lack of analogues, intermediates or relic taxa in other vertebrate or invertebrate animals. Nonetheless, speculation that an ancient piroplasm lineage evolved in invertebrates first is supported by the discovery of a piroplasm-like organism, *Cardiosporidium*, in ascidian haemocytes (Ciancio et al., 2008). Various molecular dating algorithms have also estimated the deepest node ages amongst piroplasms to range from 300 to 17 MYA (Lack et al., 2012, Schnittger et al., 2012), despite the lack of fossil records to calibrate molecular clocks. As tick ancestry is considered to be more ancient than that of most mammals, it is mooted that piroplasms evolved first in ticks and were then transmitted to mammals with subsequent radiation in rodents, grazers and predators.

## 10 Conclusions

**Fig. 10:**
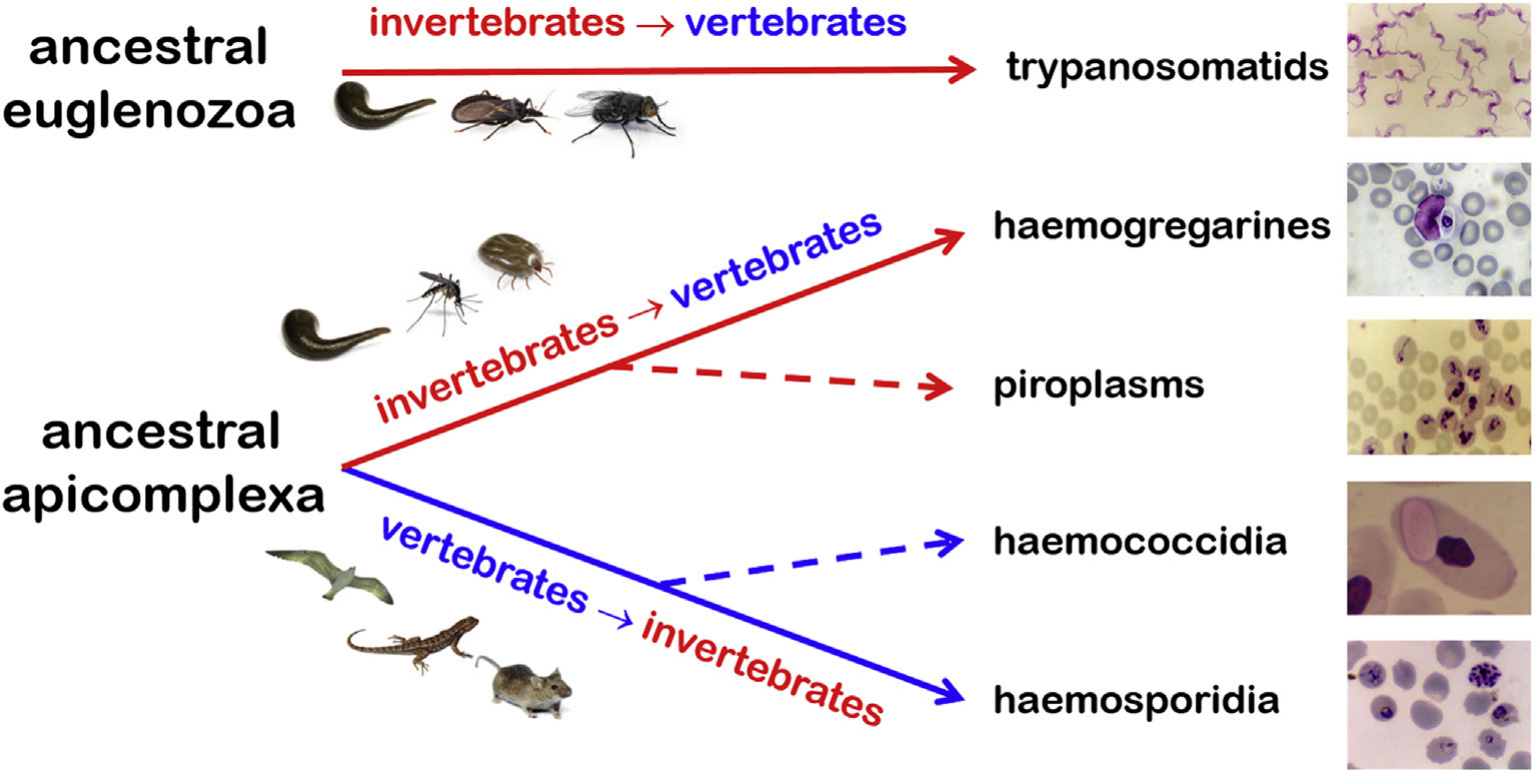
Probable evolutionary origins of haemoprotozoan parasites (solid lines = strong inferential support; dotted lines = presumptive).

When considering probable scenarios in the evolution of haemoprotozoan parasites, two main stories emerge (Fig. 10). Some groups (trypanosomatids, haemogregarines and piroplasms) apparently evolved in their vectors first, with subsequent transmission to vertebrates. Other groups (haemosporidia and haemococcidia) seem to have evolved in their vertebrate hosts first, with subsequent involvement of invertebrate vectors. These evolutionary patterns may simply reflect the availability of hosts at particular times in history, but there does appear to be a transition from aquatic to terrestrial habitats and hosts over time. The trypanosomatids and haemogregarines still have species that infect aquatic hosts and are transmitted by leeches, but the majority of haemoprotozoa infect terrestrial hosts and are transmitted by insects or ticks. It will be interesting to date the evolution of haematophagous behaviour in the different vectors and their relatives.

The evolutionary origins of vectors used by trypanosomatids predates the origins of their vertebrate hosts, often by millennia. The discovery of ‘fossilized’ trypanosomatids (*Paleoleishmania*) in mosquitoes trapped in amber during the Jurassic period ∼150 MYA (Poinar and Poinar, 2004) indicates that the ancestral parasites were present in invertebrates long before the evolution of most of their current vertebrate hosts, however, nothing is known about parasitism in any ancestral host groups. Similarly, the evolutionary origins of the vectors used by haemogregarines predates that of their vertebrate hosts, particularly for terrestrial hosts. The wealth of related parasites (gregarines and simple adeleorine coccidia) in invertebrates (and their paucity in vertebrates) tends to support the likely origins of haemogregarines in their vectors first. The piroplasms are an enigmatic and unique group previously thought to be related to the haemosporidia, but now found to be more related to some haemogregarines. Compared to other haemoprotozoan groups, the evolution of piroplasms seems to have occurred relatively recently, with some molecular clock estimates being only 17 MYA. The evolutionary origins of ticks currently used as vectors by piroplasms have been estimated to have occurred before that of their current mammalian hosts. While it may makes more sense that the piroplasms evolved in ticks first, there are no extant analogues or intermediary parasites in either ticks or mammals to assist speculation.

In contrast, the haemosporidia appear to have evolved in vertebrates first. The evolutionary origins of their current vertebrate hosts (reptiles, birds, mammals) have been estimated to have occurred around the same time as that of their current dipteran vectors (i.e. during the Jurassic Period). The discovery of ‘fossilized’ haemosporidia after that period (*Paleohaemoproteus* in a midge trapped in amber from the Cretaceous Period ∼100 MYA (Poinar and Telford, 2005) and *Plasmodium* in a mosquito in amber from the Paleogene Period ∼35 MYA (Poinar, 2005)) suggests that infected vertebrates were present for these haematophagous invertebrates to feed upon. Haemosporidia parasitize reptiles, birds and mammals, but different parasite genera utilize different vectors (black-flies, louse-flies, midges and mosquitoes). Any studies on co-evolutionary patterns need to consider tri-partite associations involving parasites, vertebrate hosts and invertebrate vectors. More studies are required to determine the vectors for many other haemosporidian species (and genera!) before meta-analyses can include consideration of host specificity, vector specificity, historical zoogeography, and ecological boundaries.

The haemococcidia are quite unlike other haemoprotozoa in that they only use invertebrates as paratenic transport hosts (without parasite development) rather than definitive or intermediate hosts (with parasite development). The haemococcidia are thought to have evolved from monoxenous enteric coccidia in vertebrate hosts, but have formed sporozoites in blood cells that have been taken up by blood-feeding vectors. Subsequent transmission back to vertebrates is not typical blood-borne transmission (inoculative/salivarian or contaminative/stercorarian), but involves the ingestion of invertebrates by vertebrates (e.g. mosquitoes and leeches eaten by reptiles and amphibians). It is not known whether this is a recent or ancient strategy, but it is not widespread and only involves a few taxa. In recent years, other gut and tissue coccidia have been found to have paratenic hosts (notably *Cystoisospora* and *Caryospora*), although such hosts are not haematophagous invertebrates but rather potential prey animals further down the food chain and involved in predator-prey transmission.

Lastly, it should be remembered that any speculation about the origins of the different haemoprotozoan assemblages are derived from studies on a small number of select taxa presumed to be representative of whole groups. More comprehensive comparative studies are required to determine whether this is the case. If there is one take-home message from all these studies, it is that Nature is diverse. Given the huge diversity of host-parasite-vector interactions, it is unsurprising that parasites have evolved along many different pathways, thus confounding attempts to make broad conceptual generalizations. Many evolutionary processes have been implicated (cf. Loker and Hofkin, 2015): involving micro-evolution below the species level (e.g. population structures, gene frequency and distribution, co-evolution (reciprocity), dispersal, virulence, adaptation, etc.); and larger-scale macro-evolution at or above the species level (e.g. allopatric/vicariant, peripatric or sympatric speciation, species diversity, global distribution, host specificity, etc.). Some studies have found reasonable congruence between host/parasite/vector lineages suggesting co-speciation (association by descent), but many others have found considerable complexity attributed to frequent host-switching, host-fitting (association by colonization), duplication, divergence, radiation, isolation and extinction events. It is apparent that we need more studies on everything: more parasites (especially overlooked, cryptic or ‘non-representative’ taxa); more ‘relatives’ (including free-living and monoxenous parasitic taxa); more vertebrate hosts (under-represented, cryptic and uncommon species); more vectors (most remain unknown); more locations (broader geographical ranges); more molecular studies on more genes (to provide consensus/concatenated interpretive analyses); more bioinformatics algorithms (to handle meta-analyses, tanglegrams and molecular dating); and particularly more biology (we need to determine the vectors and developmental cycles for many more haemoprotozoan species).

## Conflicts of interest

I declare there are no conflicts of interest with respect to the submitted manuscript.
